# Bleeding Follicular Conjunctivitis due to Influenza H1N1 Virus

**DOI:** 10.1155/2010/423672

**Published:** 2010-10-26

**Authors:** Maria Jesus Lopez-Prats, Empar Sanz Marco, Juan Jose Hidalgo-Mora, Salvador Garcia-Delpech, Manuel Diaz-Llopis

**Affiliations:** ^1^Ophthalmology Department, La Fe University Hospital, Avenda Campanar 21, 46009 Valencia, Spain; ^2^Gynaecology Department, La Fe University Hospital, Avenda Campanar 21, 46009 Valencia, Spain; ^3^Faculty of Medicine, Catholic University of Valencia, 46003 Valencia, Spain; ^4^Faculty of Medicine, University of Valencia, 46010 Valencia, Spain; ^5^CIBER of Rare Diseases, Biomedical Network Research Centre on Rare Diseases (CIBERER), 46010 Valencia, Spain

## Abstract

Influenza H1N1 or A virus is a new virus serotype capable of human-to-human transmission. This infection causes a flu syndrome similar to that of seasonal influenza, with only one case of conjunctivitis described and no clinical details or microbiological confirmation. Its diagnosis is performed by PCR of pharyngeal smear of the patients affected. We report the first well-documented case in the medical literature of conjunctivitis by H1N1 virus.

## 1. Case Report

**Figure 1:**
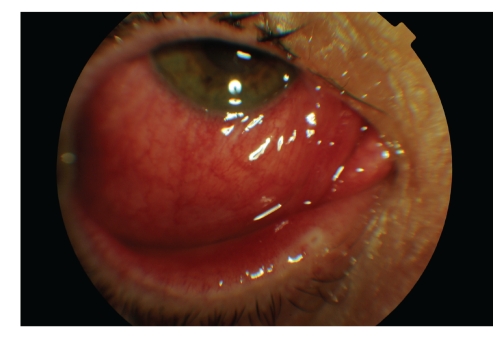
Lower chemosis and severe conjunctival hyperaemia, associated with significant eyelid oedema in both eyes.

**Figure 2:**
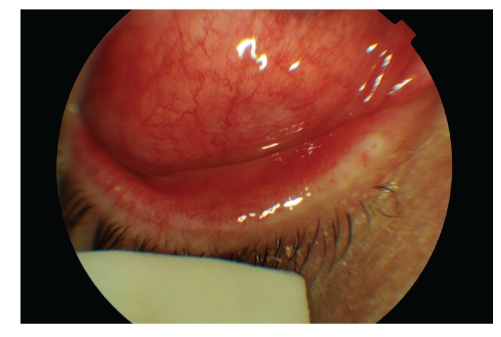
Lower chemosis and severe conjunctival hyperaemia, associated with significant eyelid oedema in both eyes.

**Figure 3:**
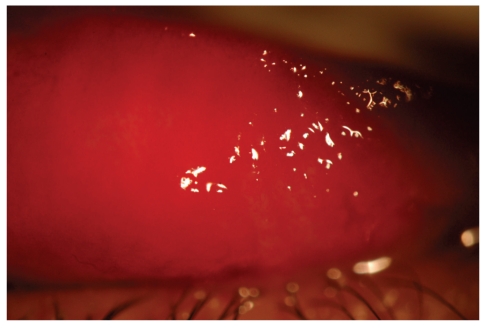
Presence of petechiae and significant upper subtarsal follicles.

**Figure 4:**
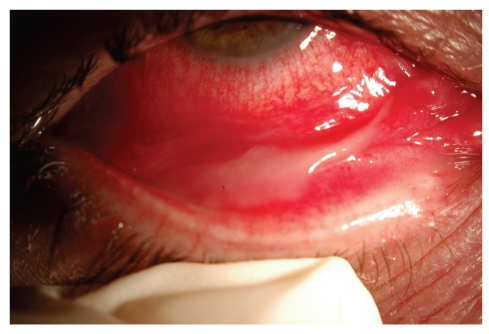
Inflammatory pseudomembranes characteristic of viral conjunctivitis.

A 45-year-old woman, five days after diagnosis by pharyngeal smear of influenza H1N1, complained of an acute unilateral conjunctivitis condition of bleeding appearance. Her examination evidenced significant eyelid oedema, severe mixed conjunctival hyperaemia and moderate chemosis, and pseudomembrane formation. A large number of subtarsal follicles and petechiae were seen (Figures 1, 2, 3, and 4).

A smear of conjunctival secretion from both eyes and study by PCR of H1N1 virus were performed detecting the presence of H1N1 virus RNA only in the affected eye, being negative in the contralateral. 

After 15 days of symptomatic treatment with topical NSAIDs and washes associated with topical ganciclovir, the condition subsided.

## 2. Discussion

In April 2009, the US CDC identified the organism responsible for the new worldwide influenza pandemic, the influenza A virus (H1N1), arising from the gene mutation of segments of two strains of an influenza virus present in swine [1].

The clinical symptoms caused by this new influenza virus are similar to those of seasonal human influenza, including high fever, cough, sore throat, generalised myalgia, headache, and asthenia, often associated with vomiting and diarrhoea. Only one case of conjunctivitis has been mentioned associated with this syndrome. Nevertheless, no information about its presentation, course, and outcome and no microbiological prove are available [2]. The CDC has developed a specific panel of real-time reverse transcriptase polymerase chain reaction (r-RT-PCR) for the specific microbiological diagnosis of the new influenza virus strain [3].

Although multiple complications have been described in association with infection by this virus, to date no microbiologically proven cases of conjunctivitis by H1N1 virus have been published. 

Viral conjunctivitis in adults is generally caused by an adenovirus, with over 50 serotypes and a high susceptibility to cause epidemics. Its most characteristic clinical signs are those discussed in our case [4]. The presence of ipsilateral preauricular adenopathy, subtarsal follicles and subconjunctival bleeding, chemosis, and formation of pseudomembranes is characteristic in severe cases. There are no specific authorised antivirals for the treatment of conjunctivitis.

In the case of our patient, it was shown how the onset of the conjunctival infection, its clinical outcome, its duration of three weeks, and the characteristics of the eye examination would be virtually identical to those of adenoviral conjunctivitis, except for the absence of preauricular node. This should lead us to consider the differential diagnosis of conjunctivitis by the H1N1 virus in patients with consistent influenza conditions, all the more given the worldwide pandemic caused by this virus which now besets us. 

In our case, the clinical outcome of the infection has been favourable, with no serious complications, and improving since the start of treatment, probably due to both the antiviral effect of topical ganciclovir and the natural course of the disease. She did not need treatment for her systemic infection. However, more cases should be described to evaluate the severity of the conjunctival infection by this new virus and the possible associated consequences.
